# Sexual Dysfunction in Systemic Autoimmune Rheumatic Diseases: Prevalence, Impact, and Management Strategies

**DOI:** 10.31138/mjr.170225.iap

**Published:** 2025-08-18

**Authors:** Monisha IN, Nilanjana Dutta, Harshwardhan Patil, Mahabaleshwar Mamadapur, Veerendra Arakalavadi Guruswamy, Pavan Kumar M R

**Affiliations:** 1 Department of Pharmacy Practice, JSS College of Pharmacy, JSS Academy of Higher Education and Research, Mysuru, India;; 2 JSS Medical College, JSS Academy of Higher Education and Research, Mysuru, India;; 3 Department of Clinical Immunology and Rheumatology, JSS Medical College and Hospital, JSS Academy of Higher Research, Mysuru, India

**Keywords:** autoimmune diseases, erectile dysfunction, physiological, quality of life, rheumatic diseases, sexual dysfunction, systemic lupus erythematosus

## Abstract

**Background:**

Systemic autoimmune rheumatic diseases (SARDs) profoundly impact patients’ quality of life (QoL) including sexual health, yet sexual dysfunction (SD) remains underrecognised in clinical care. Contributing factors include chronic pain, fatigue, joint stiffness, hormonal imbalances and psychological distress.

**Objective:**

This review evaluates the prevalence, impact, and management of SD in patients with systemic autoimmune rheumatic diseases (SARDs), including Systemic Lupus Erythematosus (SLE), Rheumatoid Arthritis (RA), Ankylosing Spondylitis (AS), Sjögren’s disease (SjD), and Systemic Sclerosis (SSc), as well as the related chronic condition Fibromyalgia Syndrome (FMS).

**Methods:**

A systematic literature review was conducted using PubMed, Web of Science, and Scopus with relevant MeSH terms. Studies reporting SD in SARDs were included, with sexual function commonly assessed via the Female Sexual Function Index (FSFI) and International Index of Erectile Function (IIEF).

**Results:**

SD prevalence ranged from 30% to 90%, varying by disease and gender. Up to 90% of women with SLE reported reduced desire and arousal. RA affected 30–70% of patients, with pain and depression as key drivers. In AS, erectile dysfunction affected 41–58% of men, influenced by age and disease duration. Over 56% of women with SjD experienced SD, often linked to vaginal dryness. SSc-induced fibrosis led to erectile dysfunction in 80% of men. FMS showed a 63% SD rate, associated with chronic pain and psychological burden.

**Conclusion:**

SD is highly prevalent yet underaddressed in SARDs. A multidisciplinary approach combining pain management, psychological support, hormonal therapy, and patient education is essential.

## INTRODUCTION

Systemic autoimmune rheumatic diseases (SARDs) encompass a diverse group of chronic inflammatory conditions, including Systemic lupus erythematosus (SLE), Rheumatoid arthritis (RA), Ankylosing spondylitis (AS), Sjögren’s disease (SjD), and Systemic sclerosis (SSc). Fibromyalgia syndrome (FMS), while not an autoimmune condition, often coexists with SARDs and significantly impacts sexual health and quality of life. These diseases significantly impact individuals across all ages and genders, leading to persistent pain, fatigue, disability, and psychological distress. Beyond the well-documented physical and functional impairments, these conditions profoundly affect sexual health, an essential yet frequently overlooked component of quality of life.^1^

Sexual Dysfunction (SD) is a complex, multifactorial condition characterised by difficulties in sexual desire, arousal, orgasm, and satisfaction, affecting both men and women. The World Health Organisation (WHO) defines sexual health as a state of physical, emotional, mental, and social well-being related to sexuality.^2^ In the context of SARDs, chronic pain, joint stiffness, fatigue, medication side effects, hormonal imbalances, and psychological distress contribute to significant impairments in sexual function.^3,4^ Women, who represent the majority of SARD patients, report reduced sexual activity, vaginal dryness, dyspareunia, and overall dissatisfaction, particularly in conditions such as SLE and SjD. In men, SD primarily manifests as erectile dysfunction, with diseases like AS and SSc showing high prevalence rates. Despite these well-recognised impacts, sexual health is often neglected in clinical practice, with both patients and physicians avoiding discussions due to time constraints, discomfort, and professional hesitation.^5^

The consequences of unaddressed SD extend beyond the physical aspects, affecting emotional well-being, mental health, self-esteem, relationship satisfaction, and overall quality of life. Studies have demonstrated strong associations between SD and increased rates of anxiety, depression, and reduced social functioning.^4^ Additionally, in women with autoimmune diseases, sexual health concerns intersect with reproductive health, influencing family planning and pregnancy outcomes.^6^ Despite its significant burden, SD remains under-recognised and undertreated in rheumatology practice, partly due to the absence of standardised screening tools and limited professional training in addressing sexual health concerns.^7^

**Figure 1.**
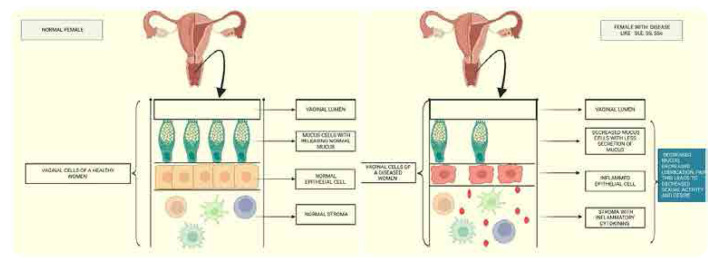
Standardised assessment strategies and multidisciplinary management approaches.

Given the substantial impact of SD on the quality of life in SARD patients, there is a critical need for greater awareness, standardised assessment strategies, and multidisciplinary management approaches (**Figure 1**). This review aims to evaluate the prevalence, impact, and management strategies of SD in individuals with SARDs, with a focus on identifying contributing factors, highlighting gaps in clinical practice, and proposing potential interventions to improve sexual health outcomes. Addressing this issue requires a comprehensive, patient-centred approach that integrates rheumatology, psychology, gynaecology, urology, and sexual health counselling, ensuring that sexual well-being becomes an essential component of holistic patient care.

## METHODS

This review employed a systematic approach to identify and synthesise existing literature on sexual dysfunction (SD) in systemic autoimmune rheumatic diseases (SARDs). The methodology adhered to rigorous search, selection, and extraction protocols to ensure comprehensive and relevant coverage of available evidence. The primary objective was to assess the prevalence, contributing factors, and management strategies of SD across major SARDs. The process involved detailed screening, eligibility assessment, and data synthesis as outlined below.

## SEARCH STRATEGY

A comprehensive literature search was conducted to identify relevant studies on SD in systemic autoimmune rheumatic diseases (SARDs). The search encompassed primary sources such as MEDLINE (2014–present) via the PubMed interface and secondary sources, including Embase and Scopus, without any time restrictions. A systematic search strategy was employed, incorporating key terms such as “Systemic Lupus Erythematosus (SLE),” “Rheumatoid Arthritis (RA),” “Ankylosing Spondylitis (AS),” “Sjögren’s disease (SjD), “Systemic Sclerosis (SSc),” and “Fibromyalgia Syndrome (FMS),” alongside other relevant autoimmune diseases.

Additional sources such as Google Scholar, PubMed Central, and ScienceDirect were manually screened to supplement database findings to ensure a comprehensive review. Identified studies underwent a multi-step selection process, beginning with title screening to remove irrelevant studies. This was followed by abstract and full-text evaluations, applying exclusion criteria that included review articles, editorials, duplicates, non-English publications, and studies lacking relevant outcome data.

The review included peer-reviewed articles, primarily original research, systematic reviews, and meta-analyses, specifically investigating sexual dysfunction in systemic autoimmune rheumatic diseases. Data extraction involved collecting key study characteristics, treatment outcomes, and safety profiles, followed by a detailed analysis to identify trends and significant findings. Additionally, selected review articles were cross-referenced to procure further relevant studies, ensuring the breadth and depth of the literature examined. This structured approach facilitated the identification of significant scientific findings that form the foundation of this review article.

## SCLERODERMA

Scleroderma (Systemic sclerosis) is an autoimmune condition that leads to varying degrees of fibrosis, vascular occlusion, and inflammation. It is usually confined to the skin. Scleroderma in later stages may lead to fibrosis and progressive organ dysfunction. It causes fibrosis and tightening of the skin and the mucosal layer of the vagina in females causing changes in mucosal secretion in the vagina, leading to difficulties in lubrication and arousal and causing painful intercourse, thus affecting the sexual function in females. Patients with scleroderma also have decreased stamina and exercise capacity, which may also alter their sexual behaviour. Studies have shown that the scleroderma mainly affects the sexual function of males due to fibrosis and tightening of the skin in the reproductive part of males, causing erectile dysfunction leading to SD. Literature indicates that most studies evaluating SD involve men more commonly, as erectile dysfunction has been noted in around 80% of affected patients due to vasculopathy and cavernous fibrosis.^8^ Female Sexual Function Index (FSFI) in patients with SSc was decreased in those with Raynaud’s phenomena, vaginal dryness or discomfort. It was found that scleroderma women were similar to normal women concerning SD. However, psychosocial health was an important contributing factor to SD**.**

## SJÖGREN’S SYNDROME

Sjögren’s disease (SjD) is a systemic autoimmune disease that affects mainly the exocrine glands and the epithelium (mucosal layer). It results in dryness of organs with mucosal surfaces, such as the vagina, mouth, and eyes (**Table 1**). Certain viruses cause inflammation in the epithelium and decreased secretion of mucosa in the vagina; it leads to difficulty and unable to maintain arousal, causing difficulty in females to perform sexual intercourse. This may be aggravated in perimenopausal women due to a gradual decline in sex hormones, leading to more dryness and atrophy of tissue, causing pain and loss of sexual desire. Peripheral neuropathy may also be attributed to causing SD as there is insufficient vasodilation, which may cause inadequate lubrication.^9^ Recently, a survey from the National Sjögren’s Foundation has demonstrated that sex life is the most often negatively affected aspect of QoL in patients with SjD.^10^ Quantitative studies on sexual functioning in female patients with SjD measured SD using a generic instrument; the Female Sexual Function Index (FSFI) questionnaire.^8^ Based on this FSFI, women with SjD showed impaired sexual function compared with healthy individuals, as reflected by significantly lower scores in the domains of desire, arousal, orgasm, lubrication, and pain. Furthermore, patients experienced more sexual distress and were less often sexually active than controls. A study by Nimwegen showed that 56% of the patients had SD and that women with pSS have greater levels of SD in several areas, including desire, arousal, orgasm, lubrication, and discomfort, when compared to healthy controls. Vaginal dryness was a predominant factor in SD, leading to increased usage of lubricants by women as compared to normal individuals. Besides vaginal dryness, physiological and psychological factors like exhaustion, depressive symptoms, unhappy relationships, and a diminished mental quality of life had a role to play in SD.^4^ Karakus found that the sexual functionality of patients with pSS depended on the duration of the disease; the greater the duration, the lesser the sexual functionality. Vaginal dryness as a result of pSS was said to be the cause of more significant inflammation and atrophy in cervical smears of those with pSS as compared to controls.^12^ Similar findings were reported in cervical cytology, colposcopic examination, and HPV DNA testing by Cirpan et al.^13^ Another study by Rebel et al. (2025) 14 conducted a qualitative study on sexual experience and functioning in patients with SjD, revealing that SD is prevalent in both female (64%) and male (67%) SjD patients. The study identified six key themes influencing sexual experience: chronic stress, SjD symptoms, invisible suffering, illness perception and coping, partner relationship quality, and the taboo nature of sexuality.

**Table 1.** Various mechanisms of sexual function of patients with SARD.

**Condition**	**SLE**	**AS**	**SjD**	**SSc**	**Fibromyalgia**
Mechanism	Idiopathic	Idiopathic	**Vaginal mucosal dryness** leads to inflammation in the epithelial tissues. **Reduced mucosal secretion** results in inadequate vaginal lubrication. **Inflammation and decreased secretion** contribute to discomfort and difficulties in sexual arousal.	**Fibrosis and skin tightening** in females cause structural changes in the vaginal mucosal layer. **Altered mucosal secretion** leads to difficulties in vaginal lubrication. **Reduced lubrication and arousal difficulties** contribute to discomfort and pain during intercourse.	**Low libido** due to chronic pain, fatigue, and hormonal imbalances contributes to reduced sexual desire. **Nerve dysfunction** and decreased blood flow can impair genital sensitivity and response, leading to difficulties with arousal and orgasm. **Pelvic muscle tightness** and increased pain perception can make intercourse painful, especially in women.

Table 1 outlines the key mechanisms contributing to sexual dysfunction in various systemic autoimmune rheumatic diseases, including factors such as inflammation, fibrosis, hormonal imbalances, and neurological impairment. SARD: systemic autoimmune rheumatic diseases; SLE: Systemic Lupus Erythematosus; AS; Ankylosing Spondylitis; SjD; Sjögren’s Syndrome; SSc; Systemic Sclerosis.

These themes highlight the complex interplay of physical, psychological, and social factors affecting sexual health in SjD. The research also emphasised that patients with SjD report an unmet need for support and treatment for sexuality-related symptoms. A study by Isik et al. (2024)^15^ investigated gynaecological symptoms and their effects on sexuality in primary Sjögren’s disease (pSjD) and secondary Sjögren’s disease (sSjD). The study found that vaginal and vulvar dryness and dyspareunia were significantly more common in SjD patients, particularly those with pSjD, compared to healthy controls. Gynaecological and musculoskeletal symptoms were shown to negatively impact sexuality in SS patients, with this effect being more pronounced in pSjD. These findings underscore the importance of assessing gynaecological symptoms and their effects on sexual health in SjD patients. Furthermore, research highlights that sexual experience in SjD patients is shaped by more than just physical dysfunction. Key factors include chronic stress, symptoms of SjD, invisible suffering, illness perception, partner relationships, and societal taboos. Many patients report an unmet need for support and treatment of sexuality-related symptoms within healthcare. Addressing these concerns as part of holistic care is essential, and future research should consider the full spectrum of patient experiences, including male SD in SjD.

## SYSTEMIC LUPUS ERYTHEMATOSUS

SLE is an autoimmune disease with protean manifestations. SLE affects individuals belonging to the child-bearing age group. SLE affects sexual activity. SLE potentiates ovarian failure or ovarian insufficiency; the mechanism behind this is idiopathic (**Table 1**). It also causes vaginal dryness, difficulty in lubrication, and maintaining orgasm during intercourse. Few similar studies have proved that women with SLE have difficulty in having intercourse and physical and psychological weaknesses, making it difficult to conceive; if conceived, they are considered to have high-risk pregnancies. Other studies have suggested that compared to normal individuals, Individuals with SLE report higher rates of decreased desire, arousal, lubrication, orgasm, and increased pain. Most studies investigating these issues have utilised the Female Sexual Function Index (FSFI). This 19-item self-reported questionnaire assesses sexual function across six domains: desire, arousal, lubrication, orgasm, satisfaction, and pain. Pinto et al.^5^ (**Table 2**) conducted a study on the correlation between sexual functioning and SLE in premenopausal women, based on these domains revealed that nearly all patients experienced SD in at least one domain. Additionally, 91.1% of patients had dysfunction in three or more domains, with the most affected areas being desire, arousal, and lubrication.^5^ This was further explored by Pena, who conducted a similar study on Mexican women with SLE and obtained identical results.^16^ A survey by Jin et al. showed that males had a higher predilection towards developing SD than females.^17^

**Table 2.** Overview of various existing studies on SARD.

**SLE review of literature**					
**Author**	**Study design**	**Population**	**Objectives & Comparison**		**Outcome**
Benzeeta et al., 2019	Observational cross-sectional study	112 Premenopausal married women diagnosed with SLE.	Assessing sexual function using the Female Sexual Function Index scoring (FSFI) questionnaire. Healthy women without SLE who serve as controls		Impaired sexual function was observed in 60.7% (68) of the women, with approximately 90% (101) reporting issues in desire, arousal, and lubrication compared to healthy controls.
M. Schmalzing et al., 2020	Case-control study	88 female patients with systemic lupus erythematosus from two German tertiary university hospitals.	Assessing sexual function using the Female Sexual Function Index scoring (FSFI) questionnaire and Qualisex as a screening test. Healthy women without SLE who serve as controls		Of the 88 SLE patients, 67.0% (59) were sexually active. Only 14.8% had ever discussed sexual issues with their physician. A SD score of 25.53 (±5.06) was observed in the patients.
Griselda Serna Pena et al., 2021	Case-control study	102 The study involved Mexican women aged 18 to 60 years with a diagnosis of SLE, as well as a control group of 156 healthy women matched for age.	Assessing sexual function using the Female Sexual Function Index scoring (FSFI) questionnaire. Healthy women without SLE who serve as controls		102 women with SLE compared with 121 healthy, sexually active women without SLE reported that 63.7%(65) of women had less sexual activity and 90%(92) women had impaired in domains like desire and arousal compared to healthy women
Maryam Shami et al., 2023	Randomised controlled study	101 This clinical trial was conducted with 101 married women aged 18–49, suffering from SLE, residing in Tehran.	Sexual counselling using the EX PLISSIT model on enhancing sexual function in married women with SLE.		Before the intervention, there was no significant difference in the sexual function scores between the two groups. Out of 110 women, 90%(99) had impaired sexual activity in domains like desire and arousal compared to healthy females.
**RA Review of Literature**						
R B Saad et al.	Cross-sectional study	71 Tunisian women diagnosed with rheumatoid arthritis (RA)	Assessing sexual function using the Female Sexual Function Index scoring (FSFI) questionnaire. Healthy women without SLE who serve as controls		Women with RA had a 49.3% frequency of female SD. Arousal (3.27 ± 1.5), orgasm (3.77 ± 1.5), and desire (2.92 ± 1.3) were the most affected areas.
S Yoshino et al.	Cross-sectional study	112 female rheumatoid arthritic (RA) patients.	Assessing sexual function using the Female Sexual Function Index scoring (FSFI) questionnaire. Healthy women without SLE who serve as controls		91 (81%) indicated that Most patients experienced a decline in sexual desire, which resulted in less frequent and less satisfying sexual encounters; impaired hip and knee joints made it challenging to assume sexual positions; and patients with unsatisfactory sexual relationships reported a decrease in their spouses' demand for sexual encounters as well as a reduction of the frequency of their orgasms.
Y El Miedany et al.	cross-sectional study	231(includes both males and females) Of this, 140 were females Patients with chronic rheumatoid arthritis who visit the outpatient rheumatology clinic	Assessing sexual function using the Female Sexual Function Index scoring (FSFI) questionnaire. Healthy women without SLE who serve as controls		SD was reported by 64 out of 140 women (45.7%). A significant correlation was found between erectile dysfunction in men and factors such as age, disease activity, pain score, cardiovascular disease, fatigue score, intramuscular steroid injection, and tender joint count. A strong correlation was also observed between SD and women.
**AS Review of Literature**						
M. Gözüküçük et al.	Cross-sectional study	99 Ankylosing spondyloarthritis in women	Assessing sexual function using the Female Sexual Function Index scoring (FSFI) questionnaire. Healthy women without SLE who serve as controls		The group with ankylosing spondylarthritis had considerably greater scores for speculum discomfort and clicheal & labial atrophy. The Ankylosing Spondylarthritis group had/have considerable scores and mostly lower FSFI than the control group. The FSFI did not show any significant differences between the Ankylosing Spondylarthritis subgroups.
Biyu Shen et al.	cross-sectional study	The study involved 103 patients with ankylosing spondylitis (78 males and 25 females). Chinese patients with ankylosing spondylitis	Assessing sexual function using the Female Sexual Function Index scoring (FSFI) questionnaire. Healthy women without SLE who serve as controls		Compared to healthy people, AS patients had significantly more severe SD. Two measures of body image disturbance (distress and social functioning impairment) and the Ankylosing Spondylitis (AS) Disease Activity Index were linked to worse partner relationships.
**SjD Review of Literature**						
Çağlar Yildiz et al.	Case-Control Study	31 Patients with SjD IN Outpatients of the rheumatology department	Assessing sexual function using the Female Sexual Function Index scoring (FSFI) questionnaire. Healthy women without SLE who serve as controls		Although no significant differences were found in endocervical culture results, a significantly higher proportion of pSjD patients reported a positive history of vaginal infections compared to controls (n=26, 83.9% vs. n=7, 25.9%, respectively). Cervical smear evaluations of the patient group showed more inflammation and atrophy compared to the control group. The average FSFI score for the patient group was significantly lower than that of the control group (18.9±9.9 vs. 25.1±5.1, respectively). Additionally, the patient group scored significantly lower in five of the six FSFI domains: lubrication, orgasm, satisfaction, pain, and excitement.
Minan Al-Ezz et al.	A controlled analysis	65 The patient diagnosed with SjD	A comparative cross-sectional study was conducted to evaluate sexual function using the Female Sexual Function Index (FSFI) in 65 female patients with primary Sjögren's syndrome (pSjD) and 62 sex-matched control participants. 65 female patients with pSjD and 62 sex-matched control participants		The study shows that SD is more prevalent in female pSjD patients, affecting 82.1%, with fatigue playing a greater role than vaginal dryness. Oral and vaginal dryness appeared independent. SD mainly impacted social quality of life, with higher depression rates, underscoring the need for comprehensive care involving sexual health assessments, fatigue management, and mental health support.
**Fibromyalgia Review of literature**						
Osman Barut et al.	Case-Control Study	46 Patients who were diagnosed with fibromyalgia according to the American College of Rheumatology (ACR) criteria and 35 healthy control aged between 20–55.	The goal of this study was to evaluate sexual function in women with fibromyalgia syndrome and compare its prevalence and quality to that of the general population. Healthy women without fibromyalgia who serve as controls		Both groups were comparable in terms of age, maritalduration, number of children, family type, and educationallevel. Women with fibromyalgia had an average duration ofsymptoms of 5.62 (3.45) years, an FIQ score of 59.8 (12.2), and a VAS score of 7 (2). The FSFI score for group 1 was 13.2 (8.1), while group 2 had a score of 32.1 (5.2). Group 1exhibited significantly higher mean scores on the FIQ, BDI, and VAS compared to group 2. Additionally, a significantnegative correlation was found between the total FSFI scoreand both FIQ and BDI scores in group 1.
Ioanna Minopoulou et al.	Meta-analysis	68 females diagnosed with a SARD	-	-	This meta-analysis results showed that the overall prevalence of SD was 63%, with an average Female Sexual Function Index (FSFI) total score of 19.7. Among sexually active females, SD prevalence was slightly lower at 60%, while the FSFI total score was higher at 22 Among different systemic autoimmune rheumatic diseases (SARDs), the highest SD prevalence was observed in women with SjD and systemic sclerosis.

Table 2 summarises key studies investigating sexual dysfunction in systemic autoimmune rheumatic diseases, highlighting study design, sample size, population, objectives, comparisons, and outcomes.

FSFI: Female Sexual Function Index; SD: sexual dysfunction; SLE: Systemic Lupus Erythematosus; RA: Rheumatoid Arthritis; AS: Ankylosing Spondylitis; SS: Sjögren’s Syndrome; SSc: Systemic Sclerosis.

## RHEUMATOID ARTHRITIS

Rheumatoid arthritis **(**RA) which is predominantly a musculoskeletal disease affecting middle-aged females, is also known to cause extra-articular manifestations like ILD, subcutaneous nodules, and sicca syndrome. Patients with RA experience not only inflammatory symptoms such as pain and synovitis but also psychological challenges, including depression, sleep disturbances, and body dysmorphic disorder, which subsequently causes SD. SD occurs in about 30–70% of patients,^18^ manifesting as decreased libido, sexual dissatisfaction, erectile dysfunction, vaginal dryness, and increased pain during intercourse.^19^ Hill et al. reported that exhaustion and pain caused problems during sexual activity for 56% of RA patients. ^20^ Reduced desire in 50%–60% of RA patients, decreased frequency of sexual activity in up to 73% of patients, aversion to sexual contacts, and decreased sexual satisfaction with time in comparison to normal patients are all signs of diminished sexual drive.^21^ Gutweniger et al. reported that female RA patients’ perceptions and sense of self were impacted by their morning stiffness. Additionally, compared to females with lower levels of morning stiffness, those with higher morning sickness incidences were more self-conscious about their bodies and felt less satisfied with their sexual experiences.^22^ El Miedany^23^ (**Table 2**) conducted a study on 231 rheumatoid arthritis patients and found that 53.8% of men and 45.7% of the most common indications of SD were orgasm, arousal, and satisfaction issues in women and erectile dysfunction in males.^1^ Quality of Life and Sexuality in Rheumatology (Qualisex) is a recognised instrument for evaluating how RA affects sexual health and quality of life.^23^ The ten questions address a variety of sexual health topics, including lubrication, arousal, desire, orgasm, and satisfaction.^24^ Men with RA did not have a significantly different rate of erectile dysfunction compared to their age-matched comparators. No major concerns have been reported about RA treatment and the higher risk of SD. In general, both conventional disease-modifying antirheumatic drugs (DMARD) and biological DMARD (bDMARD) are safe treatments regarding sexuality side effects. Only a few cases with loss of libido/impotence after methotrexate have been reported in the literature, especially in males.

## AXIAL SPONDYLOARTHRITIS

Axial spondyloarthritis (axSpA) is a chronic inflammatory condition primarily affecting the sacroiliac joints, axial skeleton, and thoracic cage.^25^ The Assessment of SpondyloArthritis International Society (ASAS) classifies axSpA into two forms: radiographic axSpA, commonly referred to as ankylosing spondylitis (AS), and non-radiographic axSpA (nr-axSpA),^26,27,28^ which lacks definitive radiographic sacroiliitis and is considered an early stage of AS.

Beyond musculoskeletal limitations, individuals with axSpA frequently experience fatigue, sleep disturbances, anxiety, depression, and stress, all of which significantly impact the quality of life (QoL) and sexual health.^29,30^ Ankylosing spondylitis typically manifests in early adulthood, affecting individuals during their most productive years and imposing significant challenges on both personal and societal levels.

Beyond its well-documented impact on mobility and daily functioning, recent studies highlight that SD is a major concern for individuals with ankylosing spondylitis, often arising from pain, spinal stiffness, and psychological distress, including depression. Addressing the broader impact of axial spondyloarthritis, particularly its effects on sexual health, is crucial for improving overall patient well-being and quality of life.^20,31–33^ While research on this topic has predominantly focused on male patients, findings indicate that men with AS experience considerably lower sexual function scores compared to healthy controls. However, the impact of non-radiographic axial spondyloarthritis (nr-axSpA) on sexual function remains unclear for both men and women.^33^

Female sexual function is a multifaceted process influenced by psychological, physiological, and personal factors.^34^ A healthy sexual response in women requires a complex interplay between the neurological, vascular, and hormonal systems (**Table 1**). Despite the significant impact of rheumatic diseases on sexual health, many patients, particularly women, tend to overlook or deprioritise this aspect of their well-being.^35^ Research conducted by Liu et al. indicates that men may experience a greater degree of SD than women, likely due to a longer disease duration, elevated inflammatory markers such as C-reactive protein (CRP), and more pronounced radiographic abnormalities. 36 Certain studies have shown that erectile dysfunction is more prevalent in individuals with ankylosing spondylitis, with prevalence rates ranging from 41% to 58%, influenced by age, disease duration, and severity.^37,38^ Shen B, Zhang A, et al.^31^ reported a significant prevalence of SD among Chinese patients with ankylosing spondylitis, with factors like disease severity, pain, and psychological distress contributing to the issue. The findings emphasise the need for a holistic approach to treatment that addresses both physical and psychological aspects of sexual health in this population. Liu et al. conducted a systematic review and meta-analysis to assess the impact of ankylosing spondylitis on sexual function. Their findings indicated that SD, particularly erectile dysfunction, is highly prevalent, ranging from 41% to 58% in AS patients, with both physical and psychological factors contributing to the condition. This comprehensive analysis underscores the need for early screening and management of sexual health issues in AS patients. ^39^ Chung et al.^29^ performed a population-based study to examine the association between erectile dysfunction and ankylosing spondylitis. The study found a significant correlation between AS and an increased risk of ED, highlighting the importance of addressing SD as part of the overall management of AS patients.^37^ Santana conducted a cross-sectional study on patients diagnosed with AS. She evaluated them using the International Index of Erectile Function (IIEF), which showed that AS patients had a higher incidence of ED than healthy controls.^40^ Studies have shown that women with axial spondyloarthritis (axSpA) frequently experience SD, influenced by disease activity, psychological factors such as anxiety and depression, and overall impaired quality of life (QoL). In addition to these medical and psychological aspects, several other elements—including past experiences, sociocultural background, level of education, and ongoing treatment regimens—may further impact sexual function. Despite its significant effects, SD in female patients with axSpA is often overlooked in routine clinical care. Recognising and addressing these concerns as part of a comprehensive treatment approach is essential to improving both sexual well-being and overall quality of life in affected individuals.

## FIBROMYALGIA

Fibromyalgia syndrome (FMS) is a chronic disorder marked by persistent pain, disrupted sleep, and cognitive impairments. SD in FMS stems from a complex interplay of neurological hypersensitivity, hormonal disruptions, chronic pain, psychological distress, and fatigue. In their case-control study, Osman Barut et al.^41^ (**Table 2**) discovered that women with fibromyalgia syndrome (FMS) had significantly worse sexual function (FSFI score of 13.2 ± 8.1) than healthy controls (FSFI score of 32.1 ± 5.2, P < 0.05). The fibromyalgia impact questionnaire [FIQ] mean scores were 59.8 ± 12.2, the visual analog scale [VAS] mean scores were 7 ± 2, and the Beck Depression Inventory [BDI] mean scores were considerably higher in the FMS group (P < 0.05). In the FMS group, a negative connection was seen between FSFI scores and both the Fibromyalgia impact questionnaire and Beck depression scores.^41^ Ioanna Minopoulou et al. (**Table 2**) conducted a meta-analysis where results showed that the overall prevalence of SD in fibromyalgia was 63%, with an average Female Sexual Function Index (FSFI) total score of 19. Among sexually active females, SD prevalence was slightly lower at 60%, while the FSFI total score was higher at 22 (95% CI: 20.8–23.1, I^2^ = 93%) among different systemic autoimmune rheumatic diseases.^42^ A holistic, multidisciplinary approach incorporating pain management, psychological care, hormonal evaluation, and pelvic therapy can aid in improving sexual function. Low libido due to chronic pain, fatigue, and hormonal imbalances contribute to reduced sexual desire. Nerve dysfunction and decreased blood flow can impair genital sensitivity and response, which causes arousal and orgasm Difficulties. Pelvic muscle tightness and increased pain perception can make intercourse painful, especially in women.^43^

## EVALUATION OF SEXUAL DYSFUNCTION

**Figure 2.**
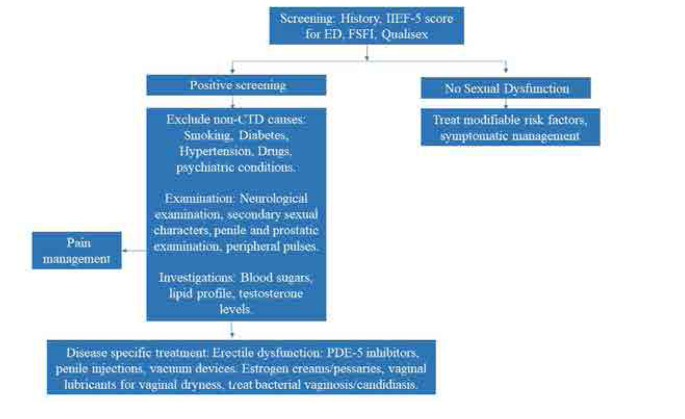
Outline of a patient-centred approach to SD evaluation.

Assessment of SD in patients with SARDs relies on standardised, patient-friendly tools. The most commonly used instruments include the **Female Sexual Function Index (FSFI)**, a 19-item questionnaire that evaluates six domains of female sexual health—desire, arousal, lubrication, orgasm, satisfaction, and pain—and the **International Index of Erectile Function (IIEF)**, a 15-item tool assessing erectile function, sexual desire, orgasmic function, and satisfaction in men. In clinical practice, it is important to create a supportive environment that encourages patients to openly discuss sexual concerns. This can be facilitated through visual aids such as anatomical diagrams, educational videos, and simple, respectful communication. Patients should receive information on how their disease may affect sexual function, including physical symptoms, psychological stressors, and medication-related factors.^38^ **Figure 2** outlines a patient-centred approach to SD evaluation, while **Table 3** presents an overview of various assessment tools applicable to both male and female patients.^44^

**Table 3.** Evaluation of SD for females and males.

**Gender**	**Tools or various measures to assess Sexual function**	**No of questions**	**Assessment area/domains**
Males	Index of premature ejaculation	10	Sexual satisfaction, control, distress
	IIEF (International index of erectile function)	15	Erectile function, Orgasm, desire, intercourse, satisfaction
Females	FSFI (Female sexual function index)	19	Desire, arousal, lubrication, orgasm, satisfaction, pain
	Qualisex questionnaire	10	Desire, satisfaction, Orgasm, arousal, pain, psychological factors like anxiety, depression, loss of self esteem
	SFQ (Sexual function questionnaire)	26	Desire, arousal, lubrication, sensation, orgasm, enjoyment, dyspareunia, partner relationship
Both	ASEX (Arizona sexual experience scale)	5	Drive, arousal, penile erection/vaginal lubrication, orgasm, satisfaction

Table 3 presents commonly used assessment tools for evaluating sexual dysfunction in both males and females, including their domains of assessment and number of questions.

FSFI: Female Sexual Function Index; IIEF: International Index of Erectile Function; ASEX: Arizona Sexual Experience Scale; SFQ: Sexual Function Questionnaire.

## TREATMENT

Management of SD involves taking a holistic approach to treating the patient’s symptoms and disease and addressing psychological concerns. To appropriately treat SD in patients with rheumatic conditions, disease activity, joint deformities, and depression, endocrinological and drug-related aspects should be thoroughly dealt with, accompanied by individualised recovery strategies, psychological support, and lifestyle modifications to optimise their overall health and effective outcomes. Risk factors like diabetes, pain, systemic hypertension, and smoking cessation should also be addressed. Patient education on simple measures like resting before sexual activity, vaginal lubricants to reduce dryness, anti-inflammatory drugs to reduce joint tenderness and stiffness, pelvic exercises, Yoga to improve SD can also prove effective. Establishing communication with patients and encouraging them to discuss sexual problems may seem embarrassing to both parties, but it is an essential component of the management of SD.

Clinicians are encouraged to assess not only the physical aspect but also the impact of SD on everyday relations and social participation. They should receive training, which could be sensitive and challenging to discuss.^42^ If an individual with SARD faces challenges related to sexual health, an open conversation can help identify the underlying causes of their intimacy issues. Tailoring a treatment plan to address these concerns is essential. Research has shown that sexual function can improve when SARD patients receive proper sexual education. In a country where gaps in sex education may lead to confusion or misconceptions, ensuring that these patients receive the proper guidance is vital. Additionally, effective management of disease activity is crucial, as it is closely linked to better sexual health and reduced pain, ultimately enhancing overall sexual function. Providing proper guidance on the use of analgesics, muscle relaxants, and timing doses to align with desired sexual activity can be very beneficial.

**Figure 3.**
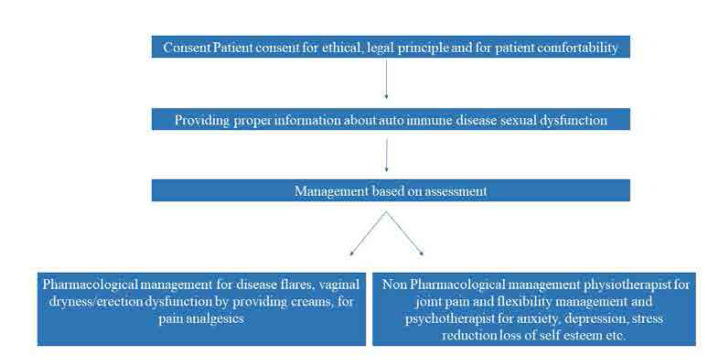
Overview of the standard management approach of SD in rheumatic disease.

A combination of medications, psychotherapy, and stress management can help address issues like depression, anxiety, and concerns related to sexual desire. It is also important to consider the mental health of a patient’s partner, with appropriate treatment for any issues they may be facing. For those experiencing endocrine dysfunction, hormone replacement therapy can be helpful. Lubricants and oestrogen creams are effective options for alleviating vaginal dryness. It is crucial to consider the possibility of common, idiosyncratic, or rare drug-induced sexual side effects where relevant. Addressing the patient’s concerns about how medications might affect their sexual function is essential, and shared decision-making between the patient and healthcare provider is often key to achieving the best outcomes.^45,46^ **Figure 3** gives an overview of the standard management approach of SD in rheumatic disease.^44^

## DISCUSSION

SD is quite common among rheumatic disease patients. However, it is not well understood and is not adequately treated. While it can affect either partner, a higher prevalence is seen in females, at around 40%.^47^ Besides the pathogenesis of SARD, increasing age, disease progression, and associated conditions like depression and fibromyalgia also play a role in SD. Since SD can be iatrogenic as well, it is important to consider the possibility of drug-induced SD in patients suffering from depression. Management of SD should involve a holistic approach covering the pharmacologic and psychologic aspects of the condition. The PLISSIT approach—Permission, Limited Information, Specific Strategies, and Intensive Therapy—is being contemplated to address this problem.^48^ Permission comprises investigating patients’ sexual function-related problems; limited information entails attempting to determine the cause through information gathering. Particular solutions would include specific interventions to address the underlying causes of SD, such as stress management, hormone replacement, current pharmaceutical management, and symptomatic treatment, such as vaginal dryness. Intensive therapy would be indicated if all these methods failed and involved referral to a specialist for treatment. Additionally, patient education regarding symptomatic management or healthy sexual practices has also been shown to improve SD.^6,49^ Along with specific therapy for SD, associated comorbidities like hypertension, diabetes, pain, and smoking should also be addressed.

This review provides a comprehensive, disease-specific analysis of SD across multiple systemic autoimmune rheumatic diseases (SARDs), addressing critical gaps in existing research and clinical practice. Unlike previous studies that focus on individual diseases, this work integrates findings across SLE, RA, AS, SjD, SSc, and FMS, identifying both shared and disease-specific patterns in SD prevalence, impact, and contributing factors. It highlights that SD is not merely a consequence of physical symptoms but is influenced by a complex interplay of disease activity, psychological distress, hormonal imbalances, medication side effects, and sociocultural factors. While much of the existing literature primarily examines male erectile dysfunction, this review places a stronger emphasis on female SD, particularly in SLE, SjD, and SSc, where vaginal dryness, dyspareunia, and decreased libido significantly affect quality of life. Additionally, the review underscores the lack of standardised screening and intervention strategies for SD in rheumatology practice, advocating for a multidisciplinary approach involving rheumatologists, gynaecologists, psychologists, sex therapists, and physiotherapists to improve patient care.

Findings from multiple studies confirm the high prevalence of SD in SARDs, with significant associations between SD and disease-related factors such as chronic pain, fatigue, joint stiffness, depression, anxiety, and hormonal imbalances. Recognising SD as an essential component of disease management is critical for improving patient outcomes and quality of life. A comprehensive, multidisciplinary approach is necessary to address these challenges effectively, ensuring patients receive holistic care. Future research should prioritize female SD, develop standardised assessment tools, and explore targeted treatment strategies to improve sexual health outcomes in individuals with SARDs. The prevalence and impact of SD across these conditions are summarised in (**Table 4**), further emphasising the substantial burden of SD and the need for dedicated clinical attention.

**Table 4.** Prevalence and impact of SD in systemic autoimmune rheumatic diseases.

**Autoimmune Disease**	**Prevalence of SD**	**Impact of SD**	**Additional Factors/Details**	**Reference(s)**
Systemic Sclerosis (SSc)	Women: 69% Men: 80% (erectile dysfunction)	High levels of SD Reduced lubrication in women, erectile dysfunction in men	Fibrosis and vascular dysfunction contribute to SD	Minopoulou et al.^42^
Sjögren's Syndrome (SjD)	64% of female, 67% of male participants reported SD Over 56% of women 74% women	Impacts pleasure, satisfaction, and sexual ability Vaginal dryness, dyspareunia, itching, and reduced sexual desire are important findings Negative impact on sexuality	Complex interplay of physical, psychological, and social factors Gynaecological and musculoskeletal symptoms affect sexuality Patients report an unmet need for support	van Nimwegen JF et al.^4^
Systemic Lupus Erythematosus (SLE)	Up to 90% of women, 18% of men	Reduced sexual desire, arousal difficulties, vaginal dryness, difficulty achieving orgasm in women Impaired sexual function	Sexual function impaired independently of disease activity, chronic disease damage, or pharmacological treatment	García Morales et al.^50^ Moghadam ZB et al.^51^ Jin Z et al.^17^
Rheumatoid Arthritis (RA)	30–70% of patients	Decreased libido, sexual dissatisfaction, and painful intercourse	Pain, fatigue, and depression are major contributing factors	Saad et al.^19^
Axial spondyloarthritis (axSpA)	Predominantly ranging from **41% to 58%**	Erectile dysfunction	Linked to disease severity and inflammatory burden	Sieper et al.^30^
Fibromyalgia Syndrome (FMS)	Around 63%	Various SD, such as decreased libido, difficulty achieving or maintaining an erection, vaginal dryness, and pain during intercourse Negative impact on sexual function	Chronic pain, fatigue, and psychological distress are contributing factors Complex syndrome involving many different factors	Bair MJ et al.^52^

Table 4 provides an overview of the prevalence, impact, and contributing factors of sexual dysfunction across different systemic autoimmune rheumatic diseases.

SSc: Systemic Sclerosis; SjD: Sjögren’s Syndrome; SLE: Systemic Lupus Erythematosus; RA: Rheumatoid Arthritis; axSpA: Axial Spondyloarthritis; FMS: Fibromyalgia Syndrome; SD: sexual dysfunction.

## CONCLUSION

Recognising the impact of autoimmune and rheumatic diseases on sexual health is essential for providing comprehensive patient care. A proactive approach that includes a thorough assessment, identifying contributing factors, and tailored management strategies can significantly improve a patient’s quality of life and overall satisfaction with care. Collaboration among healthcare professionals, including rheumatologists, psychologists, sex therapists, and physical therapists, is crucial to addressing SD effectively.

In systemic autoimmune rheumatic diseases (SARDs), female SD remains an underrecognised and under-treated issue despite its high prevalence and significant effect on well-being. Sexual health must be integrated into routine clinical discussions, with validated assessment tools used to identify and manage SD. Enhancing awareness and fostering open patient-clinician communication is a fundamental step toward patient-centred care.

Currently, there are no standardised, highly effective therapeutic solutions for female SD in SARDs. Clinical trials are urgently needed to explore appropriate interventions and support healthcare providers in managing this complex issue. By prioritising sexual health within rheumatology and autoimmune disease care, clinicians can contribute to improving patient outcomes, fostering holistic well-being, and addressing a critical yet often neglected aspect of quality of life.

## AUTHOR CONTRIBUTIONS

All authors contributed significantly to this study in accordance with ICMJE criteria. MIN was responsible for the conception and design of the study, as well as drafting the manuscript. ND contributed to data acquisition, literature review, and manuscript writing. HP performed data analysis and interpretation and drafted and critically revised the manuscript. MM was involved in manuscript drafting, critical revision, review, and editing. VAG provided final approval of the manuscript. PKM contributed to the critical revision of intellectual content and approved the final version. All authors have read and approved the final manuscript and agree to be accountable for all aspects of the work. All authors have made substantial contributions to the conception, design, analysis, and interpretation of this work. Each author has been actively involved in drafting and revising the manuscript for important intellectual content and has approved the final version for submission. This work adheres to the International Committee of Medical Journal Editors (ICMJE) authorship criteria, and all co-authors take full responsibility for the integrity and accuracy of all aspects of the study.

## CONFLICTS OF INTEREST

The authors declare no conflicts of interest related to this work.

## DISCLAIMER

This manuscript is original and has not been copied, published, or submitted elsewhere in whole or in part. All text, data, and graphics presented in this work are the author’s own, and any external sources used have been appropriately cited.

AI-based tools were utilised solely for language refinement, grammar correction, and structural improvements but not for content generation, data interpretation, or scientific analysis. The final manuscript has been thoroughly reviewed and edited by the authors to ensure accuracy and originality.

## FUNDING STATEMENT

No funding was received for the publication of this article.
